# Potential functions of microRNAs in starch metabolism and development revealed by miRNA transcriptome profiling of cassava cultivars and their wild progenitor

**DOI:** 10.1186/s12870-014-0355-7

**Published:** 2015-02-04

**Authors:** Xin Chen, Jing Xia, Zhiqiang Xia, Hefang Zhang, Changying Zeng, Cheng Lu, Weixiong Zhang, Wenquan Wang

**Affiliations:** The Institute of Tropical Bioscience and Biotechnology (ITBB), Chinese Academy of Tropical Agricultural Sciences (CATAS), Haikou, 571101 PR China; Key Laboratory of Biology and Genetic Resources of Tropical Crops, Ministry of Agriculture, Haikou, 571101 PR China; Institute for Systems Biology, Jianghan University, Wuhan, 430056 China; Department of Computer Science and Engineering, Washington University in St. Louis, St. Louis, Missouri, MO 63130 USA

**Keywords:** MicroRNA, Target Gene, Wild Progenitor, Cassava (*Manihot esculenta* Crantz)

## Abstract

**Background:**

MicroRNAs (miRNAs) are small (approximately 21 nucleotide) non-coding RNAs that are key post-transcriptional gene regulators in eukaryotic organisms. More than 100 cassava miRNAs have been identified in a conservation analysis and a repertoire of cassava miRNAs have also been characterised by next-generation sequencing (NGS) in recent studies. Here, using NGS, we profiled small non-coding RNAs and mRNA genes in two cassava cultivars and their wild progenitor to identify and characterise miRNAs that are potentially involved in plant growth and starch biosynthesis.

**Results:**

Six small RNA and six mRNA libraries from leaves and roots of the two cultivars, KU50 and Arg7, and their wild progenitor, W14, were subjected to NGS. Analysis of the sequencing data revealed 29 conserved miRNA families and 33 new miRNA families. Together, these miRNAs potentially targeted a total of 360 putative target genes. Whereas 16 miRNA families were highly expressed in cultivar leaves, another 13 miRNA families were highly expressed in storage roots of cultivars. Co-expression analysis revealed that the expression level of some targets had negative relationship with their corresponding miRNAs in storage roots and leaves; these targets included *MYB33*, *ARF10*, *GRF1*, *RD19*, *APL2*, *NF-YA3* and *SPL2*, which are known to be involved in plant development, starch biosynthesis and response to environmental stimuli.

**Conclusion:**

The identified miRNAs, target mRNAs and target gene ontology annotation all shed light on the possible functions of miRNAs in Manihot species. The differential expression of miRNAs between cultivars and their wild progenitor, together with our analysis of GO annotation and confirmation of miRNA: target pairs, might provide insight into know the differences between wild progenitor and cultivated cassava.

**Electronic supplementary material:**

The online version of this article (doi:10.1186/s12870-014-0355-7) contains supplementary material, which is available to authorized users.

## Background

MicroRNAs (miRNAs) are small (20–25 nucleotides) non-coding RNAs that have emerged as key players in post-transcriptional gene regulation in plants. They are generated from single-strand RNA precursors that are folded into stem-loop structures, and their abilities to bind to complementary sequences of target mRNAs results in cleavage or degradation of the target mRNAs or suppression of their translation [1-3]. Many studies have revealed important roles of miRNAs in development [4-7], adaptation to biotic and abiotic stresses and resistance to pathogen infection [8-10]. Many miRNAs are also specifically expressed during different stages of plant development and in specific plant organs or tissues [11-15]. For example, miR319 regulates transcription factors of the *TCP* family, which regulate multiple biological pathways, including hormone biosynthesis and signalling for cell proliferation and differentiation [4]; a set of miRNAs that affect plant hormone homeostasis and starch accumulation during grain filling in rice has also been identified [6].

Starch is an insoluble polymer of glucose residues produced by the majority of higher plant species, and is a major storage product of many of the seeds and storage organs produced agriculturally and used for human consumption [16]. Several studies reported that the targets of miRNA involved in the metabolism of carbon, sucrose, starch, lipid, etc. in switch grass [17], potato [18] and rice [6]. For example, miRn45-5p targeted *SP*S (sucrose-phosphate synthase) gene, miR1436 and miR1862 targeted *SS* (starch synthase) gene in rice. Cassava (*Manihot esculenta* Crantz), a woody shrub of the Euphorbiaceae, is one of the most important food crops in the world. It is remarkably productive in terms of its capacity to accumulate biomass and starch, and exhibits extraordinary environmental adaptability. Although about 169 miRNAs and 68 miRNA families have been predicted and characterised in cassava by both a computational approach [19] and small RNA sequencing [20], their potential roles in the production of biomass and starch have never been reported.

We are interested in identifying miRNAs and understanding miRNA functions in photosynthesis and starch accumulation in leaves and storage roots of cassava. Here, we addressed the issues of variation at the level of gene (mRNA) expression and regulation of expressed genes by miRNA in two plant organs (leaf and storage root). The two cassava cultivars (cv. KU50 and Arg7) and their wild progenitor (W14), which was deposited in Chinese Cassava Germplasm Garden and used in this study, have contrasting phenotypes in terms of photosynthetic capacity, starch accumulation and yield of storage roots (Table 1).

**Table 1:** **General characteristics of cultivar KU50, Arg7 and wild ancestor W14 used for mining and expression analysis of miRNAs and their target genes**

**Characteristic**	**KU50/Arg7**	**W14**
Latin name	*M. esculenta* Crantz	*M. esculenta. ssp. flabellifolia*
Code in Chinese cassava germplasm garden	MS000168/MS000580	MS000581
Collection site and time	Thailand , 2002/CIAT, Columbia, 2007-01-20	CIAT, Columbia, 2004-07-30
Number of fruits	few	many
Propagation method	stems	seeds
Photosynthetic efficiency (μmol/m^2^/s)	15.9–38.7	14.6–24.2
Storage root yield (kg/plant/yr)	3.0–10.0	0.5–1.0
Starch content of root (%)	28.0–32.0	3.0–5.0

## Results

### Identification of miRNAs in cultivars and their wild progenitor species

Six small RNA samples from leaves and storage roots of wild progenitor W14 and cultivars KU50 and Arg7 were sequenced using an Illumina Hi-Seq2000 instrument. New miRNAs were identified in cassava in our recent studies. We identified miRNAs that did not have greater than 70% homology with any other miRNAs in any of the searchable databases, and designated these as new miRNAs. Briefly, we incorporated the sequencing data to conduct a genome-wide search for putative loci with miRNA signatures (see details in Methods), and the expression of the miRNAs detected was quantified based on the genome sequence of AM560 [21], as well as annotation in miRBase (www.mirbase.org). A total of 62 miRNA families were detected, including 19 previously reported ones [22] (Table 2). Among these 62 miRNA families, the presence/absence of members in 51 families was conserved across the three genotypes. Ten new families, i.e., new-1, −4, −7, −12 to −16, −27 and −28, were expressed in two cultivars but not in W14. The majority of the detected miRNAs were transcriptionally active in all of the three genotypes, as determined by a normalised number of reads (NNRs). For example, new-11 had more than one thousands of reads in all sequencing libraries. In total, 107 conserved miRNAs belonging to 29 annotated families and 39 new miRNAs from 33 families had detectable expression on the basis of the sequencing data (Additional file 1). Moreover, to confirm the above findings, the expression levels of 36 miRNA families (24 conserved and 12 new) were validated by reverse transcription polymerase chain reaction (Additional file 2).

**Table 2:** **Discovery of miRNA families and members in wild progenitor W14 and cassava cultivars KU50 and Arg7**

**miRNA family**	**Members**					
	**W14**		**Arg7**		**KU50**	
	**Leaf**	**Root**	**Leaf**	**Root**	**Leaf**	**Root**
*156*	11	11	11	11	9	9
*159*, *162*, 393, *403*, *408*, 2111	2	2	2	2	2	2
*160*	7	7	7	7	4	4
*164*	3	4	4	4	3	4
*166*	8	8	8	8	8	8
*167*	6	6	6	6	5	5
*168*, 391, *397*, *398*, 530, 827, 2950	1	1	1	1	1	1
169	5	5	5	5	4	4
*171*	9	9	9	9	9	9
172	0	2	0	2	0	2
*319*	5	6	6	6	6	6
390	3	3	3	3	2	2
394	3	3	3	3	3	3
*395*	4	4	4	4	4	4
*396*	4	4	4	4	3	3
*399*	7	7	8	7	4	3
477	5	5	5	5	4	4
*535*	3	3	3	3	1	1
New-1, −4, −7, −27, −28	0	0	1	1	1	1
New-3, −25, −34	1	1	1	1	0	1
New-5, −6, −26	2	2	2	2	2	2
New-8 to −11, −22 to −24, −30 to −32	1	1	1	1	1	1
New-12	0	0	1	0	1	1
New-13 to −16	0	0	1	1	1	1
New-17, −33	1	0	1	1	1	1
New-18	0	1	1	1	1	0
New-19	0	1	1	1	0	0
New-20	4	4	4	4	4	4
New-21, −29	0	1	1	1	1	1

Whereas “0” indicates that no miRNA is detected in the corresponding genotype/organ, a number from “1” to “11” indicates the number of detected members of the miRNA family; new miRNAs have names in the format of “new-#”, for example, new-1.

Those miRNAs with names that are underlined were previously reported by Zeng et al. [22].

### Targets of cassava miRNAs

To get insight into or predict the functions of conserved and new miRNAs, a miRNA target search was performed to identify their putative targets (see Methods). A total of 360 loci on cassava unigenes were predicted to be targets of 26 conserved and 27 new miRNA families (Additional file 3), and the remaining 10 miRNA families had no predicted target. The number of targets of each miRNA family ranged from one to 25 (with an average of 9.4) for conserved miRNAs, and from one to seven (with an average of 2.9) for new miRNAs; the target number varied even within a miRNA family. Among conserved miRNAs, miR162 had one target, whereas six miRNA families (miR156, 164, 172, 319, 396 and 397) had more than 10 targets; among new miRNAs, five miRNA families (new-9, −10, −20, −22 and −26) had one target and four miRNA families (new-1, −7, −18 and −19) had more than five. Eight genes were targeted by at least two miRNAs; for example, MYB33 and MYB81 were targeted by miR159 and miR319, and SPL2 and SPL13b were targeted by miR156 and miR535. Many of these targets encode transcription factors; for example, they include the *SPL* (target of miR156 and miR535), *MYB* (miR159, miR319 and new-18), *HAM3* (miR171), *NAC* (miR164), *ARF* (miR160, miR167 and miR169), *TCP* (miR319), *GRF* (miR396 and miR477) and *SIGMA* (new-11) genes. Other miRNA targets that are functional genes included genes that encode the AGPase large subunit 2 (miR394), an F-box protein (miR394), RD19 (miR167), a member of the zinc finger family (miR172), *IRX*12 (miR397) and two disease resistance proteins (*NBS-LRR* class; miR396 and −30).

### Most miRNAs were highly expressed in the leaves and storage roots of cultivars

The NNR for each miRNA should be no less than 50, and if the NNR of a miRNA is less than 50 then it is considered to be silent (not expressed at a detectable level). Whereas 34 miRNAs (24 families) displayed a two-fold difference in their expression level between the cultivars and W14 (Table 3), 29 miRNAs (18 families) were highly expressed in the leaves of cultivars, including miR169e, miR2950, miR319cd, miR391, miR393ab, miR394abc, miR395abcd, miR399d, miR535b, new-4, new-6ab, new-12, new-13, new-15, new-17, new-28, new-29 and new-32. On the other hand, six miRNA families were more highly expressed in leaves of W14 than in cultivars, including miR156ijk, miR396c, miR477e, new-11, new-30 and new-34.

**Table 3:** **miRNAs that are differentially expressed between cultivars and their wild progenitor**

**Organ**	**Highly expressed in cultivars**	**Highly expressed in the wild progenitor**
**Leaf**	miR169e, miR2950, miR319cd, miR391, miR393ab, miR394abc, miR395abcd, miR399d, miR535b, new-4, new-6ab, new-12, new-13, new-15, new-17, new-28, new-29, new-32	miR156ijk, miR396c, miR477e, new-11, new-30, new-34
**Root**	miR156ijk, miR167acdef, miR169e, miR2111ab, miR397, miR399d, miR477cd, new-6ab, new-9, new-15, new-17, new-28, new-29	miR168, miR171abcdefghi, miR2950, miR393ab, miR396c, new-3, new-8, new-11, new-34

Furthermore, a total of 44 miRNAs (23 families) were differentially expressed in storage roots between cultivars and the wild progenitor. Thirteen miRNA families were highly expressed in storage root of cultivars; these included miR156ijk, miR167acdef, miR169e, miR2111ab, miR397, miR399d, miR477cd, new-6ab, new-9, new-15, new-17, new-28 and new-29. Meanwhile, ten miRNA families were more highly expressed in the storage roots of wild progenitor W14; these included miR168, miR171abcdefghi, miR2950, miR393ab, miR396c, new-3, new-8, new-11 and new-34 (Table 3).

### Confirmation of miRNA:target pairs by RLM-RACE

**Figure 1:**
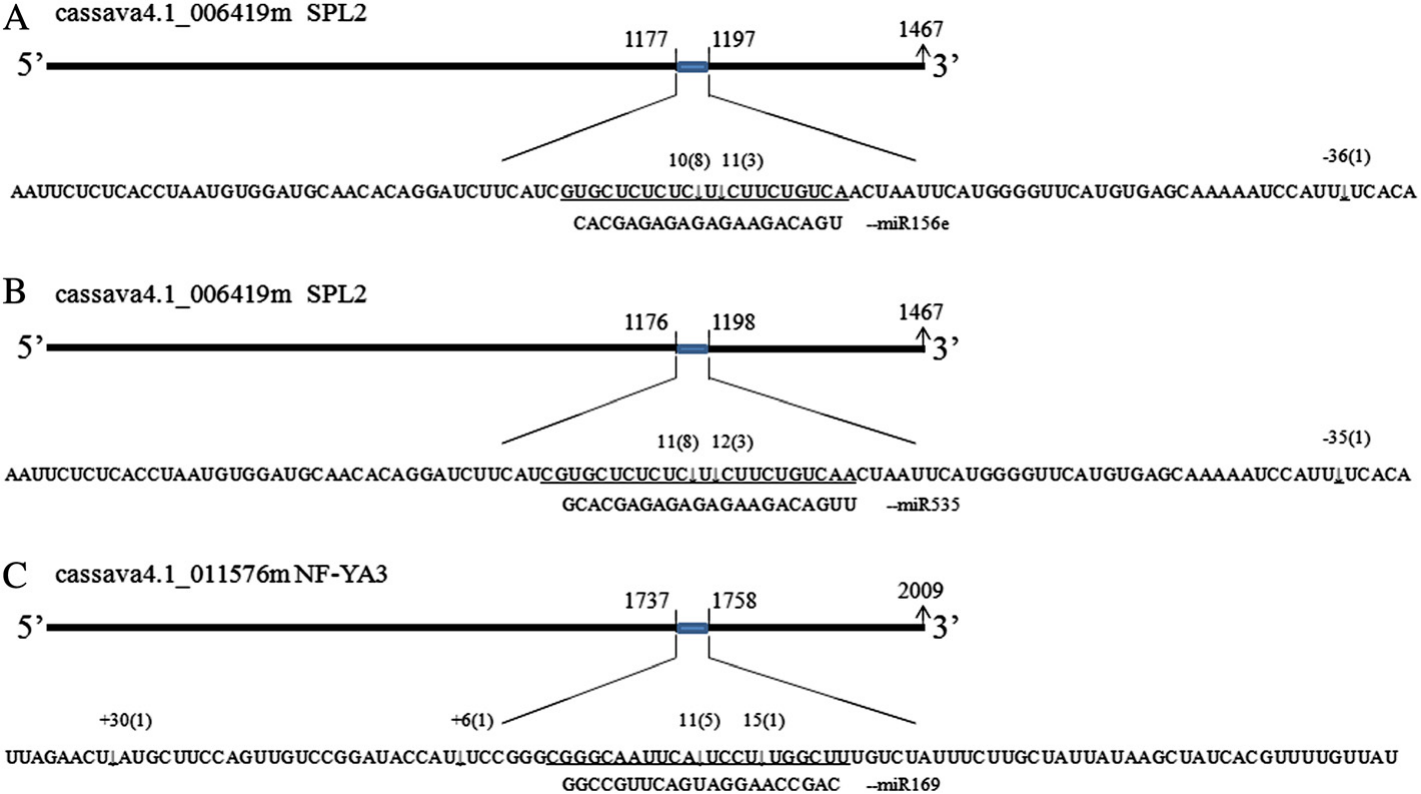
**Identification of miRNA-guided cleavage products of target genes in cassava (partial results).** The cleavage sites of selected targets as identified by 5’ RACE analysis. For each miRNA, the target sequence is shown on the top and the miRNA sequence on the bottom. The numbers indicate the fraction of cloned PCR products when PCR was terminated at different positions. **(A)** The site of cleavage of cassava4.1_006419m by miR156. **(B)** The site of cleavage of cassava4.1_006419m by miR535. **(C)** The site of cleavage of cassava4.1_011576m by miR169d.

Binding of a miRNA to the complementary sequence in target mRNA leads to mRNA cleavage. The cleavage sites located at its complementary region (CR) are direct evidence of RNA-induced silencing complex (RISC)-mediated slicing of the target mRNA. Fifteen miRNA:target pairs were selected for slicing analysis using RNA ligase-mediated rapid amplification of the 5’ cDNA ends technique (RLM-RACE). Of these, nine miRNAs (related to 12 pairs) exhibited differential expression between the cultivars and W14. The corresponding primer sets are listed in Additional file 4. Cloning and sequencing of the PCR amplicons of remnant mRNAs enabled determination of the nucleotide position when a slicing event occurred. Finally, slicing events occurred in 14 of 15 miRNA:target pairs. The cleavage sites of eight pairs were at the 10th, 11th and 12th nucleotides in the CR (Table 4 and Figure 1). miR394 sliced two targets at different sites with different efficiencies: two slicing events at the 10th nucleotide in the CR and seven events downstream of the CR for *F-box* mRNA; there were also four slicing events at the 2nd and 14th nucleotides in CR and 14 slicing events upstream or downstream of CR occurred for ADP-glucose pyrophosphorylase large subunit 2 (*APL2*) mRNA.

**Table 4:** **Confirmation of miRNA:target pairs by RLM-RACE**

**miRNA**	**Target**	**Annotation**	**Cleavage sites**		
			**Upstream**	**Complementary region**	**Downstream**
**miR156/** **miR535**	cassava4.1_006419m	*SPL2*	/	10th/11th(8), 11th/12th(3)	−35th/–36th(1)
	cassava4.1_009657m	*SPL13b*	/	9th/10th(2), 10th /11th(4), 11th/12th(1)	−1st/–2nd (1)
**miR159/** **miR319**	cassava4.1_004606m	*MYB33*	/	11th/12th(10)	−52th/–53th(1), –115th/ –116th(1)
	cassava4.1_030321m	*MYB81*	/	11th/12th(6)	−59th /–60 ^th^(1)
**miR394**	cassava4.1_021267m	*APL2*	+18th(1), +35th(3), +42th(1), +69th(2)	2nd(3), 14th(1)	−27th(1), −29th(1), −49th(1), −92th(1), −105th(1), −109th(1), −139th(1)
	cassava4.1_007038m	*F-box*	/	10th(2)	−31th(1), −52th(1), −55th(1), −59th(1), −68th(1), −75th(1), −102th(1)
**miR160**	cassava4.1_002668m	*ARF10*	/	11 ^th^(10)	/
**miR167**	cassava4.1_009942m	*RD19*	+5th(1), +25th(1), +145th(1), +196th(1)		−41th(1)
**miR169**	cassava4.1_011576m	*NF-YA3*	+30th(1), +6th(1)	11th(5), 15th(1)	/
**miR396**	cassava4.1_003731m	*GRF1*	+69th(2)	6th(1), 11th(6)	−45th(1), −60th(1), −188th(1)
**miR398**	cassava4.1_024493m	*DIR-like*	+8th(10)	/	/
**miR477**	cassava4.1_008074m	*CLB*	+37th(8)	/	/
**miR166**	cassava4.1_009671m	*GH17*	/	/	−8th(3), −16th(6), −34th(1)
**new-21**	cassava4.1_006393m	*EDR2*	/	/	−112th(6), −160th(2), −176th(1), −177th(1), −178th(1), −182th(1)

Note: As an example, 10th/11th (8) indicate that there were eight cleavage sites at the 10th or 11th nucleotide in the complementary region of cassava4.1_006419m and miR156/535; alternatively, −35th/–36th (1) indicates that there was one cleavage site downstream of the complementary region of cassava4.1_006419m and miR156/535.

Similarly, other miRNAs sliced their targets at the CR. For instance, miR160 sliced *ARF10*, with ten cleavage sites at the 11th nucleotide in the CR, and miR169 sliced *NF-YA3*, with six cleavage events at the CR. In addition, miR396 cleaved its target *GRF1* at the classic 11th site. For the remaining five pairs, cleavage sites could not be detected at the CR region, whereas they were detected up/downstream of this region. The cleavage sites of miR398:*Dir-like* and miR477:*CLB* were at the +8th and +37th nucleotides upstream of the CR, respectively. Finally, the cleavage sites of miR166:*GH17* and new-12:*EDR2* were all downstream of their CRs. Taken together, these findings indicated that many cleavage sites were not positioned at the CR or were even far away from it. Accordingly, these remnant mRNAs might have been randomly degraded mRNAs.

### Co-expression analysis of the miRNAs and their targets in leaf and storage root of cassava cultivars and their wild progenitor

**Figure 2:**
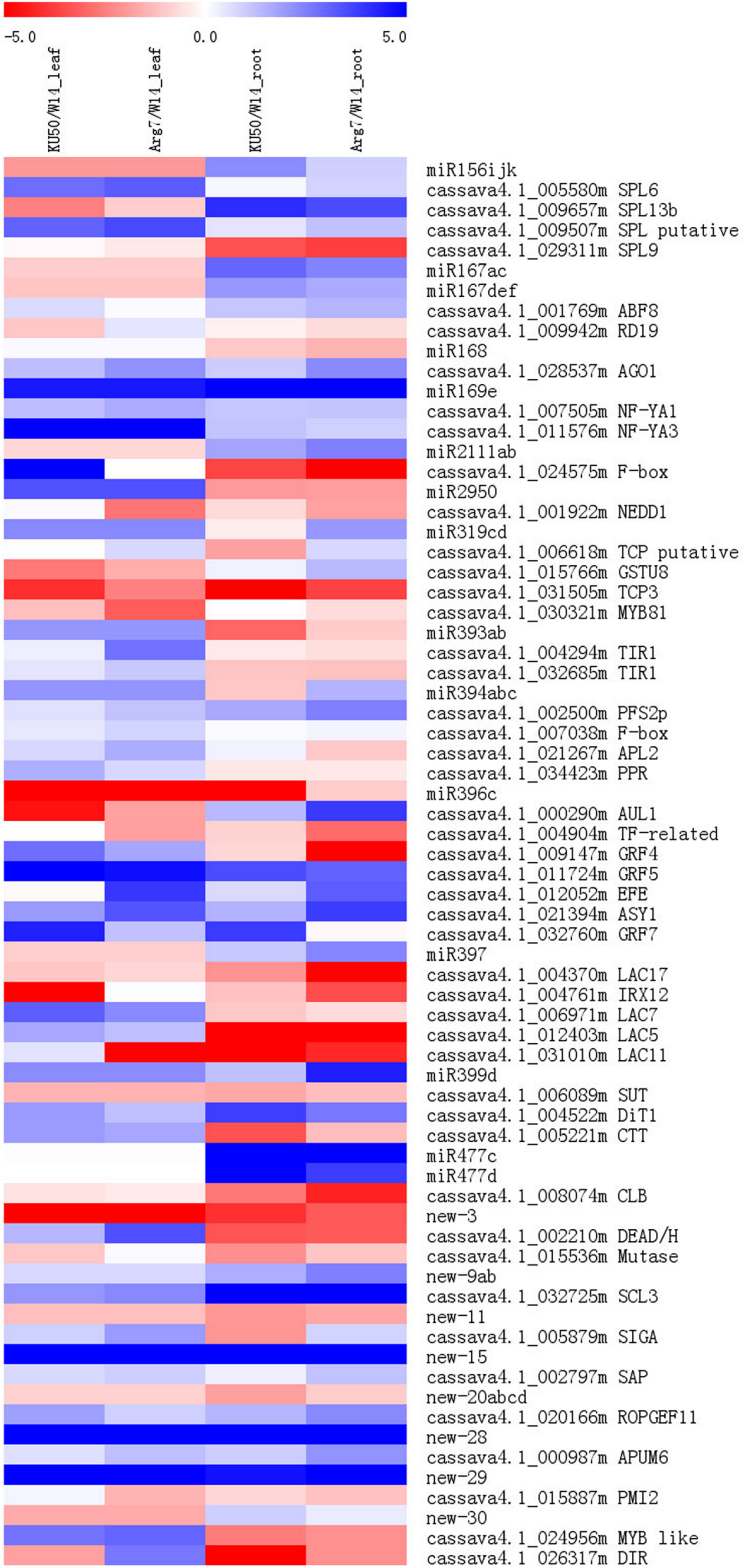
**Correlations of the levels of expression of miRNAs and their corresponding targets in leaf and storage root between cultivars and their wild progenitor.** Heat mapping was performed based on the log2- normalized expression ratio of cultivar/wild progenitor.

To further study the relationship between miRNAs and their targets, RNA-seq transcription profiling was performed to assay the expression of targets in leaf and storage root among these three *Manihot* genotypes. Co-expression analysis involved 32 miRNA families highly expressed in leaf or root (61 miRNAs) and their 87 corresponding targets. In general, the expression levels of 28 targets were negatively correlated with that of their corresponding miRNAs, which included 12 transcription factors, three plant hormone-related genes and one *SUT*. However, 22 targets showed a positive correlation with their corresponding miRNAs, including eight transcription factors, four plant hormone-related genes, one *APL2* and one *CTT* (Figure 2 and Additional file 5). In total, there were 27 transcription factors and plant hormone-related genes, and three starch biosynthesis- and sugar transport-related genes among these target genes, this might be explained by translation suppression or feedback regulation. Surprisingly, some of the miRNAs slicing their targets were confirmed, but being positively correlated with the targets; these miRNA:target pairs included miR156:*SPL13b*, miR169:*NF-YA3* and miR394:*APL2/F-box*.

**Figure 3:**
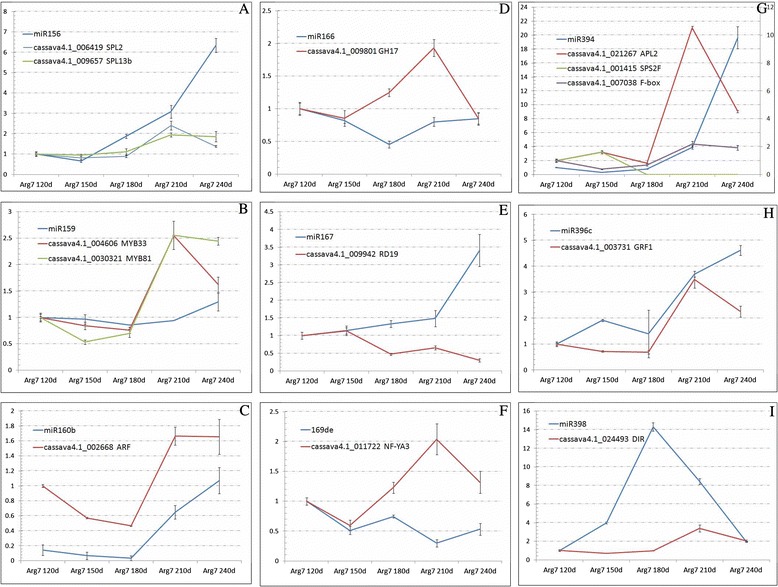
**Expression correlations of miRNAs and their targets in storage root of cultivar Arg7.** Quantification of the relative expression of miRNAs and their targets was carried out using the ^△△^CT method, with the U6 gene and *beta*-actin gene as references for miRNAs and targeted genes, respectively, and Arg7 120d as the control; Arg7 120d indicated that the storage root of Arg7 was sampled 120 days after planting, the other samples were labeled accordingly; the Y-axis means the times at which the levels of expression of miRNAs and their targets in other samples were comparable to those in Arg7 120d. **A-I**: means different miRNAs and their targets.

On the basis of the miRNA:target pair confirmation and the co-expression analysis, 13 miRNA:target pairs were chosen for further assays of their regulatory relationship by quantitative real time PCR (qRT-PCR) in Arg7 storage roots at different growth stages; the corresponding primers are listed in Additional files 2 and 6. From the results, eight miRNAs and their corresponding targets had obvious negative correlations, except for miR160b; most of these miRNAs were expressed during the later stage of growth of the Arg7 root (Figure 3). For example, miR394 targets both cassava4.1_021267m (*APL2*) and cassava4.1_000867m (sucrose phosphate synthase 2F, *SPS2F*), which are involved in starch biosynthesis. *APL2* showed low expression from 120 DAP (day after planting) to 180 DAP, increased from 180 DAP and reached a peak at 210 DAP; it then fell close to its minimum at 240 DAP. In contrast, an increase in levels of miR394 at 180 DAP, followed by a sustained increase from 210 DAP to 240 DAP, suggested that *APL2* was negatively regulated by miR394 at a later stage. The second target, *SPS2F*, maintained a relatively stable expression level at the early stage, but then fell close to zero from 180 DAP to 240 DAP; this suggested that miR394 also negatively regulated *SPS2F* (Figure 3G). However, given that there was no negative/positive correlation between miR394 and *APL2* on the basis of the RNA-seq data in roots among the three *Manihot* genotypes, the regulation might only have existed in a specific growth stage of cassava storage root.

## Discussion

The initial findings about miRNA in cassava were obtained with the aid of the castor bean genome information; specifically, 20 conserved miRNA families were reported [22], and then 17 conserved miRNA families were predicted in cassava by using *Arabidopsis* mature miRNAs as seed sequences [23]. Recently, 169 potential conserved miRNAs in cassava were identified by a computational approach, and 126 miRNAs (114 conserved and 12 new) were discovered by small RNA sequencing [19,20]. In this study, we discovered 107 conserved miRNAs (29 families) and 39 new miRNAs (33 families) by small RNA sequencing, and 36 of these families were detected again by RT-PCR. By miRNA transcriptome profiling, we identified 41 conserved miRNAs (17 families) and 19 new miRNAs (15 families) that showed differential expression between the cultivars and their wild progenitor. Recently, Xia et al. also reported 22 cassava new miRNAs, and part of them responded to chilling stress [24].

We predicted that 360 cassava unigenes were targeted by 26 conserved and 27 new miRNA families, and that 57 of them (including *MYB, SPL*, *ARF*, *NAC* and *TCP*) encode transcription factors; several similar results have already been reported in cassava [19,20] and other species [5,25,26]. Several miRNAs are involved in starch accumulation in rice [6], and five of them (miR159, miR160, miR164, miR167 and miR319) also had the same targets in cassava. In addition, two other miRNAs (miR394 and miR399) targeted *APL2* and three sugar and carbohydrate metabolism-related genes (sugar transporter, invertase and carbohydrate transmembrane transporter), which have not been reported in other species. These miRNAs might be key regulators of starch biosynthesis in cassava.

A previous study reported that miR159 regulated *MYB* mRNAs, and miR319 predominantly acted on *TCP* mRNAs in *Arabidopsis* [27]. Both miR159 and miR319 shared sequence identity at 20 of 22 nucleotides, and the expression level of miR159 was far greater than that of miR319 in two cultivars and the wild progenitor. Therefore, similar to the case in *Arabidopsis*, MYB transcription factors were mainly regulated by miR159 in cassava. MYB could function as a transcriptional activator in ABA-inducible gene expression in *Arabidopsis* [28,29], and high concentrations of ABA could suppress the expression of starch synthesis genes in maize and rice [30,31]. It was inferred that miR159 could directly or indirectly affect starch biosynthesis in cassava.

miR396 targeted 20 cassava unigenes, including five *GRF* transcription factors. *MeGRF1* (cassava4.1_003731) was verified to be sliced by miR396 in cassava. Previous studies reported that high expression level of miR396 in root tips might result in reduced expression of six *MtGRF* genes [32], and that the miR396-*GRF1/GRF3* regulatory module acted as a developmental regulator in the reprogramming of root cells during cyst nematode infection in *Arabidopsis* [33]. These findings suggested that miR396 might regulate root development in cassava.

miR169 targeted *NF-YA* family genes, and over-expression of *NF-YA5* and *NF-YA3* or down-regulation of miR169 might enhance drought stress tolerance in *Arabidopsis* and soybean [34,35]. *MeNF-YA3* was negatively regulated by miR169, and the expression level of miR169 in the wild progenitor was lower than that in cultivars, and the evidence was that the wild progenitor had stronger drought tolerance than the cultivars usually.

miR398 targeted the mRNA of a disease resistance-responsive family protein (cassava4.1_024493, *Dir-like*) in cassava, but the cleavage sites of the miR398:*Dir-like* pair were not positioned within the CR: ten cleavage sites were all at the 8th nucleotide upstream of the CR. Given that the miRNA-guided cleavage occurred quite precisely at the 10th or 11th nucleotide from the 5’ end of the miRNA in CR [24,36,37], this might be a surprising phenomenon that is difficult to explain.

## Conclusion

Using next-generation sequencing technology, we carried out miRNA transcriptome and transcriptome profiling of two cultivated cassava and their wild progenitor. A total of 107 conserved miRNAs (29 families) and 39 new miRNAs (33 families) were identified, and most miRNAs were highly expressed in the cultivars. Of the 360 unigenes predicted to be the targets of 53 cassava miRNA families, 14 unigenes were confirmed. In addition, co-expression analysis between miRNAs and their targets was performed on the basis of the miRNA transcriptome and transcriptome profiling of leaves and storage roots; the expression levels of 28 targets were negatively correlated with that of their corresponding miRNAs. In conclusion, the differential expression of miRNAs between cultivars and their wild progenitor, together with our analysis of GO annotation and confirmation of miRNA:target pairs, might provide insight into how the wild progenitor was domesticated to cultivated cassava.

## Methods

### Plant materials

Two cultivars of the cultivated species *Manihot esculenta* Crantz (KU50 and Arg7) and W14, a subspecies of *Manihot esculenta spp. flabellifolia*, were used in this study. Both KU50 (a cultivar that is extensively planted in South East Asia) and Arg7 (a cultivar from Argentina) presented with higher photosynthesis and higher storage-root yield and starch content of storage roots. This distinguished them from W14, a native of Central Brazil, which had a lower rate of photosynthesis and very low storage root yield and starch content of the storage root. *Manihot esculenta spp. flabellifolia* was previously proposed to be the progenitor of cultivated cassava [38-40]. All three genotypes were grown in an experiment field in Haikou, China. Leaves and roots of these three genotypes were sampled at 150 DAP for sequencing of small RNAs and characterisation by RNA-seq. The roots of Arg7 were sampled on 120 DAP, 180 DAP, 210 DAP and 240 DAP for expression profile analysis of miRNA and targets by real-time PCR, and miRNA-target pair confirmation by RLM-RACE.

### Small RNA extraction and Solexa sequencing

Small RNA samples of leaves and roots from the above three *Manihot* genotypes were extracted by using an miRNA isolation kit (Bioteke, Beijing, China) in accordance with the manufacturer’s instructions. Small RNAs of fewer than 30 bases were isolated from these miRNA samples, and linked with a pair of Solexa adaptors to their 3’ and 5’ ends; then, the sample was reverse-transcribed into cDNA and amplified using the adaptor primers. The double-stranded miR-cDNAs were sequenced using Illumina’s Solexa Sequencer in accordance with the manufacturer’s instructions (BGI Company, Shenzhen, China).

### RNA extraction and sequencing

Total RNA was extracted from leaves and storage roots using RNAplant reagent (Tiangen, Beijing, China) and purified using RNeasy Plant Mini Kit (Qiagen, Valencia, CA). The cDNA libraries for analysis using a Illumina Hiseq2000 instrument were prepared by following the protocol of Zhong et al. [41]. Six cDNA libraries of leaf and storage root were sequenced, and the sequenced reads were aligned to the cassava genome draft (http://www.phytozome.net/cassava) using TopHat and Cufflinks [42] and annotated using KEGG [43]. The fragments per kilobase per million reads (FPKMs) were used to normalise gene expression counts for each transcript. Transcripts with FPKM <3 were considered to be so rare as to not be expressed at all, as suggested for a study on white lupin [44].

### Identification of conserved and new miRNAs in wild and cultivated species

1. Conserved miRNA
  Mature plant miRNA sequences from miRBase (http://www.mirbase.org/) were aligned to the AM560 genome (http://www.phytozome.net/cassava). We retrieved the flanking genomic sequences around completely matched loci, with different upstream and downstream lengths, to form possible precursors of candidate miRNAs with the RNAfold program [45]. We chose those sequences with folding structures that have at least 18 bp in matched regions, one central loop and a folding energy ≤ −18 kcal/mol. The free tails in the secondary structures were then removed. Next, we applied the MiRcheck program [46] to select sequences that have ≤4 mismatches, ≤2 bulged or asymmetrically unpaired nucleotides and ≤2 continuous mismatches in the seed regions.
2. New miRNA.

We searched for new miRNAs in the three *Manihot* genotypes (namely, W14, Arg7 and KU50), using the corresponding small RNA-seq datasets. The newly identified miRNAs, combined with known miRNAs in *Manihot*, were then subjected to a homology search. We aligned mature and hairpin sequences of an miRNA to the cultivar-AM560 genome using the local alignment tool BLAST. We set the p-value obtained from BLAST to less than 1e-10 and manually examined the alignment to determine whether a BLAST hit was homologous to the input miRNA. We mapped the qualified reads from corresponding cultivar datasets to the identified homologous sequence using Bowtie [47] and counted the mappable reads. If no homologous sequences could be identified in a cultivar genome assembly, we then mapped reads from the sequencing datasets of the same cultivar to the input miRNA sequence, allowing two mismatches. We considered a miRNA to be not conserved in the cultivar genome assembly if both of the following criteria were met: 1) no homologous sequences identified and 2) insufficient mappable reads (fewer than 10 normalised reads) for analysis. If any of the reads mapped to the input miRNA sequences, we considered the miRNA to be conserved in the genome assembly even if we were not able to identify the homologous sequences.

### Identification of differentially expressed miRNAs

Reads that aligned perfectly to the candidate miRNA-yielding transcripts were used to compute the digital expression levels of the miRNAs. Reads that mapped to multiple genomic loci were attributed to all derivative miRNAs. Read counts in each sample were normalised to adjust for sample variation. If *N*_*sample*_ is the number of qualified reads that aligned to the genome and cDNA sequences in that sample and *C* is the average value of *N*_*sample*_ of all samples, then the NNR for each miRNA in each sample is (*N*_miRNA_*C/*N*_*sample*_), where *N*_*miRNA*_ is the raw sequencing reads of the miRNA. Differentially expressed miRNAs were those that had at least two fold changes between the cultivars and their wild progenitor.

### Target mRNA prediction and miRNA: target pair validation

We used the Hitsensor algorithm [48] to predict miRNA targets in AM560, which was downloaded from http://www.phytozome.net/cassava. Hitsensor searched for miRNA complementary sites in coding regions with a modified Smith-Waterman algorithm [49]. This algorithm scores these sites by giving rewards to key sequence-specific determinants, including the seed region (12–17-nucleotides long), local-AU content around the seed region and ≤3 mismatches.

RLM-RACE Gene Racer Kit (Invitrogen, CA, USA) was used to validate the predicted interaction. An RNA adaptor was ligated to the truncated mRNA, followed by reverse transcription with polyT. The next nested PCR with two gene-specific primers and two GeneRacer 5’ forward primers was performed as described previously [22]. The amplified PCR products were gel-purified, cloned into the PMD-18T vector (Takara, Dalian, China) and sequenced.

### Quantity detection of miRNA and targets

The amount of miRNA was quantified as described previously [50]. Firstly, the small RNA samples were converted to miR-cDNA by using an RT primer pool with reverse transcriptase. Then, a specific primer pair was designed for each miRNA, after which PCR amplification with SYBR Premix Ex TaqTM kit (Takara, Dalian, China) was carried out using a Rotor-Gene 6000 machine (Corbett Robotics, Australia), with the U6 gene as a control. Quantification of the relative expression of miRNAs was performed using the ^△△^CT method. Quantification of the target was also carried out using qRT-PCR, with the *beta*-actin gene as a control. The forward and reverse primers of the target were located at the two flanks of the binding region at which the miRNA interacted with its target mRNA; the primer pairs are listed in Additional file 6.

### Supporting data

The small RNA sequencing data is deposited in Gene omnibus with accession number GSE52178. RNA-seq reads are deposited in GenBank/SRR sequence read archive under the accession codes SRR1299000, SRR1299003, SRR1299009, SRR1299006, SRR1298998 and SRR1298996.

## Additional files

Additional file 1:
**Normalised digital read counts for each miRNA in leaf and storage root of three** ***Manihot*** **genotypes.**
Additional file 2:
**Primer sets used to amplify microRNAs using qRT-PCR.**
Additional file 3:
**Functional annotation of 360 predicted targeted genes.**
Additional file 4:
**Gene-specific primers for target validation by RLM-RACE.**
Additional file 5:
**Expression correlations of 21 miRNAs and their corresponding targets in leaf and root between cultivar and progenitor.** “#” means that the slicing is confirmed by RLM-RACE between miRNA and target. Additional file 6:
**Primer pairs used to amplify targets for analysis by quantitative real-time PCR.**

## Abbreviations

DAP: Day after planting
miRNA: MicroRNA
qRT-PCR: Quantitative reverse transcription polymerase chain reaction
NGS: Next-generation sequencing
NNR: Normalised number of reads
FPKM: Fragment per kilobase per million reads
